# Deciphering Genetics Underlying Stable Anaerobic Germination in Rice: Phenotyping, QTL Identification, and Interaction Analysis

**DOI:** 10.1186/s12284-019-0305-y

**Published:** 2019-07-15

**Authors:** Sharmistha Ghosal, Carlos Casal, Fergie Ann Quilloy, Endang M. Septiningsih, Merlyn S. Mendioro, Shalabh Dixit

**Affiliations:** 1 International Rice Research Institute, Los Baños, Laguna Philippines; 2 University of the Philippines, Los Baños, Laguna Philippines; 3 Bangladesh Rice Research Institute, Gazipur, Bangladesh; 4 Soil & Crop Science, Texas A&M University, 2474 TAMU, College Station, TX USA

**Keywords:** Rice, Anaerobic germination, QTLs, SNPs, KASP

## Abstract

Anaerobic germination (AG) is an important trait for direct-seeded rice (DSR) to be successful. Rice usually has low germination under anaerobic conditions, which leads to a poor crop stand in DSR when rain occurs after seeding. The ability of rice to germinate under water reduces the risk of poor crop stand. Further, this allows the use of water as a method of weed control. The identification of the genetic factors leading to high anaerobic germination is required to develop improved DSR varieties. In the present study, two BC_1_F_2:3_ mapping families involving a common parent with anaerobic germination potential, Kalarata, an *indica* landrace, and two recurrent parents, NSIC Rc222 and NSIC Rc238, were used. Phenotyping was done under two environmental conditions and genotyping was carried out through the KASP SNP genotyping platform. A total of 185 and 189 individuals genotyped with 170 and 179 polymorphic SNPs were used for QTL analysis for the two populations, Kalarata/NSIC Rc238 and Kalarata/NSIC Rc222, respectively. A total of five QTLs on chromosomes 3, 5, 6, 7, and 8 for survival (SUR) and four QTLs on chromosomes 1, 3 (two locations), and 7 for the trait seedling height (SH) across the populations and over the screening conditions were identified. Except for the QTLs on chromosomes 5 and 8, the parent with AG potential, Kalarata, contributed all the other QTLs. Among the five QTLs for SUR, the second-largest QTL (*qSUR6–1*) was novel for AG potential in rice, showing a stable expression in terms of genetic background and screening conditions explaining 11.96% to 16.01% of the phenotypic variation. The QTL for SH (*qSH1–1*) was also novel. Considering different genetic backgrounds and different screening conditions, the QTLs identified for the trait SUR explained phenotypic variation in the range of 57.60% to 73.09% while that for the trait SH ranged from 13.53% to 34.30%.

**Electronic supplementary material:**

The online version of this article (10.1186/s12284-019-0305-y) contains supplementary material, which is available to authorized users.

## Background

The ability of rice to germinate under water is generally low. Most modern rice varieties either fail completely to germinate under water or fail to elongate the coleoptile and develop roots and shoots for further development under a long period of oxygen deprivation, resulting in partial to complete crop failure (Magneschi and Perata, 2009; Narsai et al., 2015). However, genetic variation exists among rice varieties for anaerobic germination (AG), with some landraces being able to germinate under water. These genotypes are thus the sources for studying the underlying genetics as well as the physiological mechanisms of this trait. Numerous studies in the past have tried to understand the strategies adopted by tolerant genotypes to cope with this stress. Seed longevity, seedling vigor (Yamauchi et al., 1993; Yamauchi et al., 1994; Yamauchi and Chuong, 1995; Biswas and Yamauchi, 1997; Ella et al., 2010; Septiningsih et al., 2013), seedling growth and adjustment of carbohydrate metabolism (Bailey-Serres and Chang, 2005; Ismail et al., 2009; Ismail et al., 2012), fast coleoptile elongation, fast leaf and root development (Hsu and Tung, 2015; Kretzschmar et al., 2015), and high carbohydrate reserve of seed (Al-Ani et al., 1985; Raymond et al., 1985; Ella et al., 2011) are some of the mechanisms reported underlying the trait. These processes further involve more specific mechanisms such as the continuation of carbohydrate metabolism through rapid breakdown of starch to provide energy for germination (Ella and Setter, 1999; Ismail et al., 2009; Magneschi and Perata, 2009); increased activity of enzymes such as α-amylase to facilitate starch breakdown (Guglielminetti et al., 1995; Perata et al., 1997; Hwang et al., 1999; Bailey-Serres and Chang, 2005; Ismail et al., 2009; Ismail et al., 2012); alcohol dehydrogenase (ADH, EC1.1.1.1) (Ismail et al., 2012), which is involved in the anaerobic pathway (alcoholic fermentation) of carbohydrate metabolism (Avadhani et al., 1978; Jackson et al., 1982; Waters et al., 1991; Setter and Ella, 1994; Gibbs et al., 2000); increased activity of enzymes involved in cellular expansion such as expansin for enhanced cell wall loosening (Huang et al., 2000; Choi et al., 2003; Ismail et al., 2009; Magneschi and Perata, 2009); and the increase in phytohormones such as gibberellic acid (GA) (Gu et al., 2010; Septiningsih et al., 2013).

Extensive molecular breeding efforts have also been made in the past in order to identify genes and underlying genetic mechanisms. Recently, a series of linkage mapping studies identified many QTLs with major and minor effects on AG (Jiang et al., 2004; Jiang et al., 2006; Angaji et al., 2010; Septiningsih et al., 2013; Baltazar et al., 2014). One of these QTLs, *qAG9–2* on chromosome 9, has been fine-mapped to *OsTPP7*, which is responsible for starch mobilization to drive embryo germination and coleoptile elongation (Kretzschmar et al., 2015). A recent study on RNA-Seq analysis of coleoptile elongation reported 26 genes related to membrane structure and cell walls (Hsu and Tung, 2017). Nevertheless, further efforts are needed to identify regulatory processes for traits that are unique to tolerant genotypes. The exploration of novel QTLs for tolerance of anaerobic conditions during germination is essential to understand the genetics and molecular basis of tolerance and to provide robust QTL targets for marker-assisted breeding.

The objective of this study was to identify stable and robust QTLs by exploring two BC_1_F_2:3_ populations, derived from Kalarata, an *indica* landrace with high AG, and two high-yielding rice varieties with low AG, and by using two screening methods. The identified QTLs will be the bases for advanced investigation of the genetic mechanisms underlying AG potential and for providing additional avenues for varietal development through marker-assisted breeding.

## Materials and Methods

### Mapping Population

This study was conducted using two BC_1_F_2:3_ QTL mapping populations generated by crossing Kalarata, a parent with high AG potential originating from India, with two high-yielding but susceptible recurrent parents, NSIC Rc222 (IRRI 154) and NSIC Rc238 (IRRI 156), developed by IRRI. All the parents belong to subspecies *indica*. The growth duration of NSIC Rc222, NSIC Rc238 and Kalarata is 98, 104, and 118 days, respectively. NSIC Rc222 yields an average of 5.7 t/ha to a maximum of 7.9 t/ha. Meanwhile, NSIC Rc238 is characterized by an average yield of 6.4 t/ha to a maximum of 10.6 t/ha. Kalarata on the other hand is a low yielding landrace with yields ranging between 2 and 4 t/ha. Plant height of Kalarata, NSIC Rc 222 and NSIC Rc 238 are 144, 98 and 104 cm, respectively. A population of 205 lines for each of the crosses (Kalarata/NSIC Rc222 and Kalarata/NSIC Rc238) underwent phenotypic evaluation. Further, genotypic profiles of a set of 189 and 185 lines were generated for the two populations, respectively, which were used in the study. The parents for each population were used as checks for the respective populations.

### Phenotyping and Data Collection

**Fig. 1:**
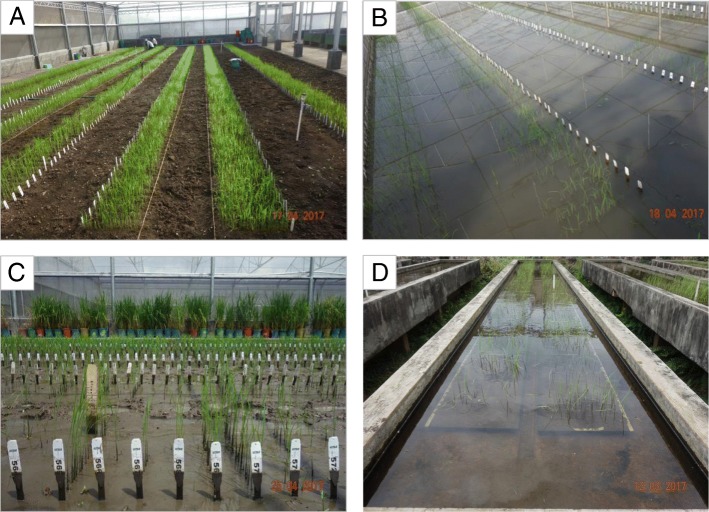
Phenotyping of the rice mapping population (BC_1_F_2:3_) in screenhouse. **a** Control experiment at 14 DAS, **b** stress experiment at 14 DAS, **c** stress experiment at 21 DAS after removing water in the screenhouse, and **d** stress experiment at 14 DAS in trays-on-table

Both populations underwent two screening conditions for the evaluation of AG: one using trays filled with garden soil placed on a concrete screening table and the other directly on the puddled soil of a screenhouse, providing more natural field conditions for the experiments. Controlled experiments were also conducted under dry direct-seeded conditions in a screenhouse (Fig. 1). The experiments were conducted during the dry season in 2017 at the experiment station of the International Rice Research Institute (IRRI), Los Baños, Laguna Philippines (14°11′N, 121° 15′E).

Dry seeds were used for the experiments. Dormancy was broken by keeping the seeds at 52 °C for 72 h in a hot-air oven. A 15X15 α-lattice design with three replications was used to conduct the experiments in all conditions.

For the table screening, seedling trays (53.3 X 38.19 X10.2 cm^3^) filled with fine garden soil were marked with a grid marker maintaining seeding depth of 1.0 cm and 15 lines per tray. Thirty seeds from each entry were sown in each line. After seeding, the lines were covered with fine garden soil. The trays were submerged carefully in concrete benches filled with 7–8 cm of water from the soil surface of the trays. This water depth was maintained for the entire experiment duration (21 days). Two measuring scales were used for each table to constantly monitor and maintain the water level.

For the screenhouse experiment, standard land preparation for wet seeding was followed. After leveling, the field was left for 24 h for the soil to settle down. The field was divided into beds and 45 cm-long rows were laid out on the beds. These rows were drawn 10 cm apart with a depth of 1 cm using a grid marker. Thirty seeds from each entry were sown in each row and were covered with 1 cm of topsoil. The field was slowly submerged with 7–8 cm of water above the soil surface and was maintained as such for 21 days. Six measuring scales were placed at each corner and in the center of the field to monitor and maintain the water depth. Water temperature was monitored daily at 7 AM, 2 PM and 6 PM in both experiments (Additional file 1: Figure S1).

For the control experiments in the screenhouse, standard land preparation for aerobic dry-soil conditions was followed. The protocol for layout and seed sowing was the same as it was in puddled soil except that the seeds were sown directly in the dry soil. After seeding, irrigation was done by overhead sprinklers. Three tensiometers were placed at equal distances inside the field to monitor soil moisture and thereby apply irrigation to maintain saturated conditions up to 21 days.

Data on seedling count and height were recorded for all the experiments. The number of seedlings that emerged above the water surface for anaerobic conditions was counted on the 14th and 21st day of the experiment and the height of five random seedlings were measured on the same days and average seedling height (SH) was determined as well. For the experiment under aerobic conditions, seedlings were also counted on the 14th and 21st day after seeding (DAS) to determine germination percentage (GER) and the height of five random seedlings were measured and averaged to determine the mean SH for each line. Additionally, data on seed pericarp color was collected for both populations after dehulling the seeds of each line. This was done through visual scoring where the line is scored as 1 if 100% of the seeds are white, 3 if 75% of the seeds are white, 5 is 50% of the seeds are white, 7 if 25% of the seeds are white, and 9 if all seeds are red. The subset of lines scored with 1 was also used for downstream analyses apart from the two individual population analyses.

### Statistical Analysis

SUR was calculated as the percentage of the number of surviving seedlings relative to the total number of seeds used. Analysis of variance (ANOVA) was conducted using PBTools V 1.4.0. and CropStat7.2.3 (http://bbi.irri.org/products). The linear mixed model described below was used for α-lattice design analysis:

$$ {\mathrm{P}}_{ijk}=\upmu +{\mathrm{R}}_i+{B}_J\left({\mathrm{R}}_i\right)+{G}_k+{\mathrm{E}}_{ijk} $$

P_ijk_ is the measurement recorded on a line, μ is the overall mean, R_i_ refers to the effect of the i^th^ replicate, B_j_ refers to the effect of the j^th^ block within the i^th^ replicate, G_k_ refers to the effect of the k^th^ genotype, and E_ijk_ refers to the error effect. For computation of means and standard error of difference (SED), the effects of replications and blocks within replications were considered as random, whereas, for the computation of variance components, the effects of genotypes, blocks, and replication were considered random. Broad-sense heritability was calculated as

$$ {H}^2=\frac{\sigma_G^2}{\sigma_G^2+{\sigma}_E^2/R} $$

Here, H^2^ stands for broad-sense heritability, ${\sigma}_G^2$ for genetic variance, ${\sigma}_E^2$ for error variance, and R for the number of replications in the experiment. Correlation among traits, the frequency distribution, and graphical visualizations were done using the packages “corrplot”, (Wei and Simko, 2017), “psych” (Revelle, 2018) and “ggplot2” (Wickham, 2016) in R (R core team, 2018).

### Genotyping

A total of 189 families from the Kalarata/NSIC Rc222 population and 185 families from the Kalarata/NSIC Rc238 population were selected for genotyping. Leaf samples were collected from a bulk of 20 plants per family and lyophilized for DNA extraction. Genotyping was done through the KASP assay genotyping platform of 1100 SNPs based on MSU Rice Genome Annotation Project Release 7 (Kawahara et al., 2013) at Straits Biotech Pte. Ltd., Singapore. KASP assays were performed using a mix containing universal FRET (fluorescence resonance energy transfer) cassettes (FAM and HEX), ROX™ passive reference dye, *Taq* polymerase, free nucleotide, and MgCl_2_ in an optimized buffer. End-point fluorescence data were visualized with a microplate reader (PHERAstar^plus^, BMG LABTECH, Germany) and analyzed by Klustering Caller software7. Reaction mixtures consisted of final volumes of 5 μL containing 2.5 μL of 2 X KASP V4.0 Mastermix, 0.056 μL of assay primer mix (12 mM of each allele-specific primer and 30 mM of common primer), and 50–100 ng of genomic DNA. An S1000 Thermal Cycler (Bio-Rad) was used with the following cycling conditions: 94 °C for 15 min, 9 cycles of 94 °C for 20S, touchdown starting at 65 °C for 60S (decreasing 0.8 °C per cycle), 32 cycles of 94 °C for 20S, and 57 °C for 60S.

### Linkage Map Construction and QTL Analysis

A linkage map was constructed based on Kosambi map function using the QTL mapping tool QTL IciMapping version4 (Meng et al., 2015). A LOD value of 3.0 was used for estimating map distance. QTL mapping was done using R/qtl ver1.40–8 (Broman et al., 2003). The function “scanone” was used to map QTLs using the Haley-Knott regression method assuming genotyping error rate of 0.01. A total of 10,000 permutations were conducted and threshold values for the 5% and 1% level of significance were determined. The percentage of phenotypic variation explained by each QTL and the QTL effect were estimated by a drop-one-ANOVA analysis implemented in fitqtl function. The total phenotypic variation explained by the QTL was calculated using a model in fitqtl function that takes into account all QTLs and their interactions. The confidence interval for each QTL was defined using a 2-LOD-support interval. The epistatic interaction was determined by the R/qtl function scantwo that was fitted to the model using MQM algorithm in R/qtl. The interactions were declared at the 1% level of significance threshold based on 1000 permutations. In order to determine whether the QTLs in this study are novel or the same as those previously reported, the relative positions of the confidence intervals and QTL peaks were compared.

QTL class analysis was performed using QTLs which were common to both populations. Lines, irrespective of the population, were classified into groups based on the presence or absence of the QTLs. SUR mean for screenhouse condition at 21 DAS for both stress and non-stress experiments were used to calculate the QTL class advantage over the mean of the two populations to determine the effect of a QTL class. Kruskal-Wallis rank sum test was performed to compare the different QTL classes across populations using “FSA” (Ogle et al., 2018) package in R (R core team, 2018). Further, Pairwise Mann-Whitney U-tests was used for post hoc analysis using “rcompanion” (Mangiafico, 2019) and “multcompView” (Graves et al., 2015) in R.

Finally, desirable donor lines were selected based on four criteria: (1) presence of the detected QTLs in varying combinations; (2) percentage survival under anaerobic conditions; (3) white pericarp color; and (4) percentage germination under anaerobic conditions.

## Results

### Phenotypic Variation and Trait Correlations

Two populations along with their respective parents as checks were analyzed for their phenotypic performance under stress and non-stress conditions. Significant differences were observed among the genotypes while no significant difference was observed between replicates. Table 1 and Additional file 1: Table S1 present the summary statistics of SUR and SH, respectively, under all environments from both populations. The sensitive parents showed low SUR and SH compared with the tolerant parent and the families for both populations. The average SUR of the parents was 42.7% to 58.2% for Kalarata, 5.1% to 10.3% for NSIC Rc238, and 0.7% to 8.6% for NSIC Rc222 under anaerobic conditions. The normal germination of Kalarata ranged from 77.6% and 89.7%, that for NSIC Rc238 was 82.2%, and that for NSIC Rc222 was 90.6%. The range of SUR of the population Kalarata/NSIC Rc238 was 0.0 to 76.1% and for the population Kalarata/NSIC Rc222 it was 0.0 to 76.7% under anaerobic conditions (Table 1). Germination under normal conditions for Kalarata/NSIC Rc238 and Kalarata/NSIC Rc222 was 68% to 94% and 64% to 99%, respectively (Table 1). Similarly, for SH, the population mean ranged between those of taller parent Kalarata and shorter parents NSIC Rc222/NSIC Rc238 (Additional file 1: Table S1). All trials showed significant differences among genotypes, with heritability (H) values ranging between 0.45 and 0.97 for both traits.

**Table 1:** Performance of Two Rice BC_1_F_2:3_ Mapping Populations of Kalarata/NSIC Rc238 and Kalarata/NSIC Rc222 Along With Respective Parents for SUR Under Anaerobic Conditions During Germination

	KALARATA/NSIC Rc238 POPULATION						KALARATA/NSIC Rc222 POPULATION					
	Survival 14 DAS			Survival 21 DAS			Survival 14 DAS			Survival 21 DAS		
	Control	Screen house	Tray-on-table	Control	Screen house	Tray-on-table	Control	Screen house	Tray-on-table	Control	Screen house	Tray-on-table
Kalarata	77.6	47.4	43.6	77.6	52.5	56.5	89.7	42.7	47.0	89.7	58.2	55.0
NSIC Rc238/NSIC Rc222	82.2	6.8	5.1	82.2	10.3	9.5	90.6	3.6	0.7	90.6	7.3	8.6
Mean	81.8	21.3	20.8	81.8	29.3	29.4	85.1	22.0	24.4	85.1	32.9	32.6
Range	68.0–94.0	0–67.3	0–63.3	68.0–94.0	0–73.3	0.4–76.1	64.0–99.0	0–63.3	0–66.7	64.0–99.0	0–76.7	0–73.3
SED	7.17	8.10	5.10	7.17	5.23	8.83	6.50	6.66	6.24	6.50	6.88	6.94
*P* value	^a^	^a^	^a^	^a^	^a^	^a^	^a^	^a^	^a^	^a^	^a^	^a^
Heritability	0.62	0.92	0.96	0.62	0.97	0.92	0.58	0.93	0.95	0.58	0.95	0.94

^a^= Significant at 0.0001% *P* levels

**Fig. 2:**
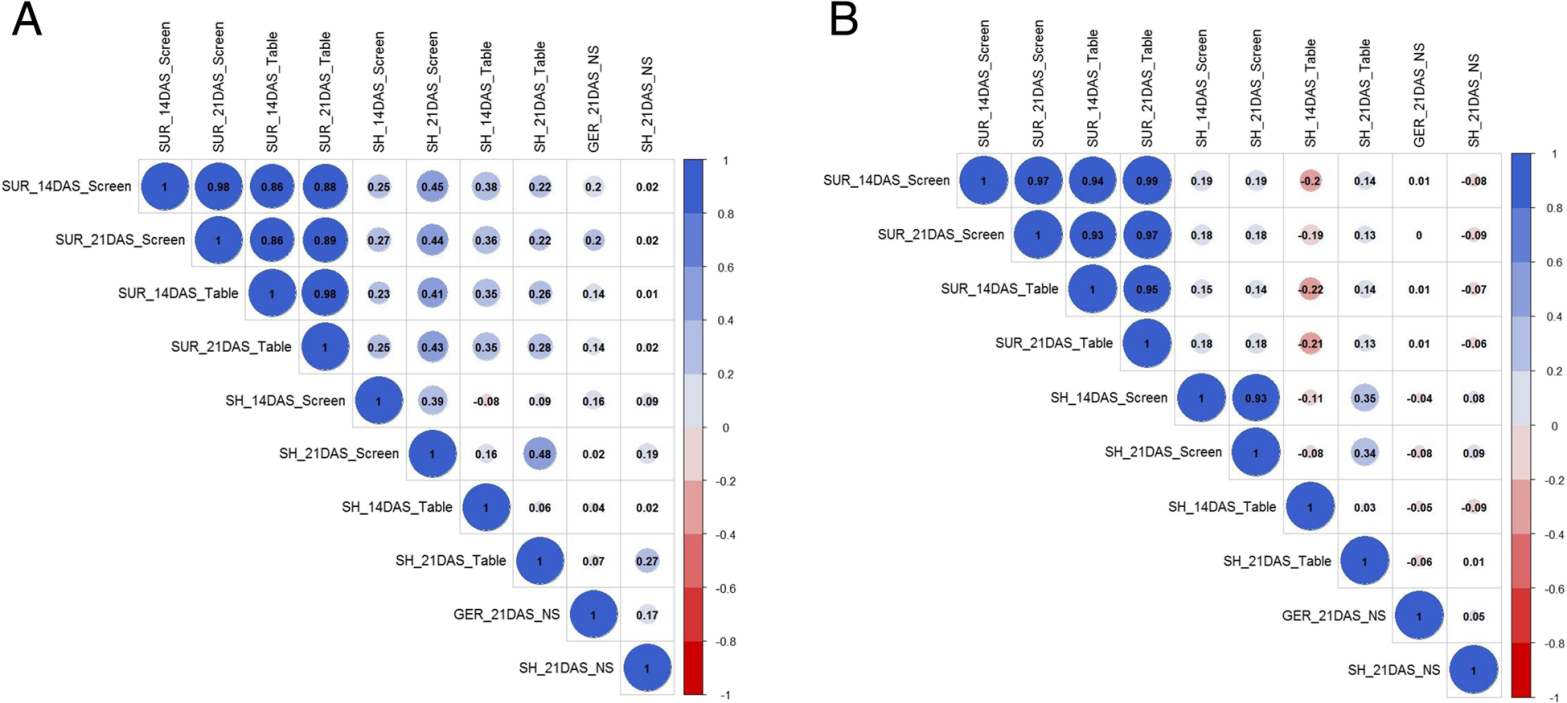
Correlation among survivability, seedling height, and germination (control) under screenhouse, tray, and control conditions (NS) for **a** NSIC Rc238/Kalarata and **b** NSIC Rc222/Kalarata populations

Continuous variation with a small skewed frequency distribution was observed for all the traits under study. A significant positive correlation was observed for SUR across screenhouse and tray-on-table screening conditions for both 14 and 21 DAS. Higher correlation was observed within each screening conditions compared to those across screening conditions for the Kalarata/NSIC Rc238 population (Fig. 2a); while this is not the case for Kalarata/NSIC Rc222 population (Fig. 2b). For example, highly significant correlation coefficients, 0.89 and 0.97, for SUR at 21 DAS in tray-on-table and in screenhouse experiments were noted for the populations Kalarata/NSIC Rc238 and Kalarata/NSIC Rc222, respectively. A positive significant correlation was also observed between SUR and SH for both populations. Non-significant correlations between germination under control conditions and SUR under AG conditions for both populations were observed, suggesting that there is no relationship between normal germination and AG traits (Fig. 2a and b). Heritability ranging from 58% to 97% was observed for seedling germination, while values ranging between 92% and 97% were observed for survival under anaerobic conditions (Table 1). This indicates the suitability of the screening protocol for the trait.

### Linkage Map and QTL Mapping

**Fig. 3:**
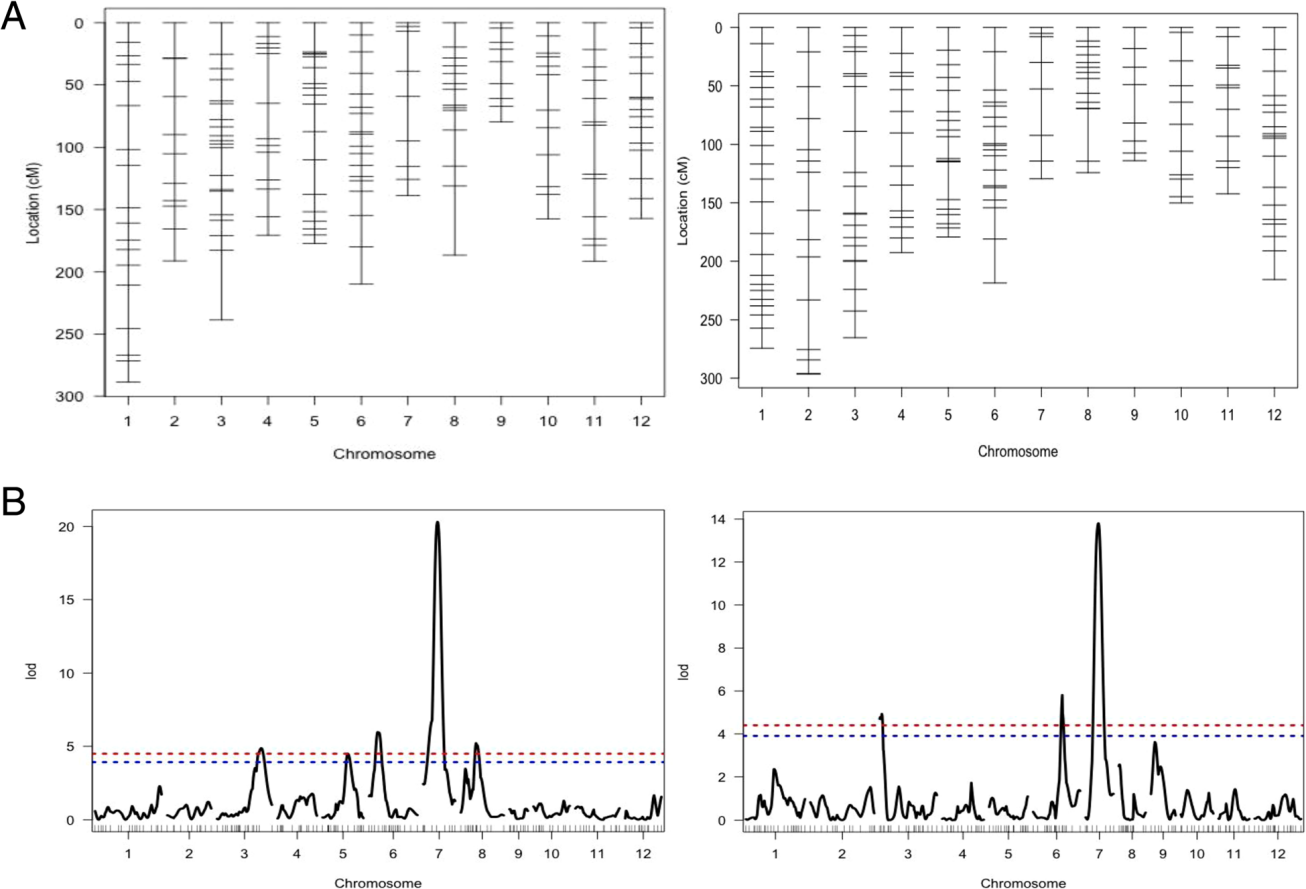
**a** Genetic map of Kalarata/NSIC Rc238 population (left side) and Kalarata/NSIC Rc222 population (right side) showing marker and QTL positions. **b** Whole-genome scan plots obtained by interval mapping for SUR at 21 DAS in screenhouse conditions for Kalarata/NSIC Rc238 population (left side), and Kalarata/NSIC Rc222 population (right side). Horizontal lines indicate the significant logarithm of odds threshold at 5% and 1% confidence levels (from the bottom to the top) based on 10,000 permutations

A total of 290 polymorphic SNPs for the Kalarata/NSIC Rc238 population and 305 polymorphic SNPs for the Kalarata/NSIC Rc222 population were identified. The linkage map was constructed with 170 evenly spaced polymorphic SNPs for the Kalarata/NSIC Rc238 population and 179 polymorphic SNPs for the Kalarata/NSIC Rc222 population (Fig. 3a). The map for Kalarata/NSIC Rc238 covered 2188.22 cM of the rice genome with an average interval of 12.87 cM while the map for Kalarata/NSIC Rc222 covered 2301.21 cM of the rice genome with an average interval of 12.85 cM. QTL analysis revealed several significant QTLs for both traits studied and most of them were consistent across the environments and across the populations as both populations involved a common parent with good anaerobic germination potential.

The QTLs were declared at the 5% level of significance threshold based on 10,000 permutations (Fig. 3b). However, most of the QTLs were found significant at the 1% level as well. The identified QTLs and their chromosomal position, peak markers, additive effects, and contributions in explaining phenotypic variations are presented in Tables 2 and 3 for the populations Kalarata/NSIC Rc238 and Kalarata/NSIC Rc222, respectively. Whole-genome scan plots for the two populations for the two traits are presented in Fig. 3b.

**Table 2:** List of QTLs Detected for Anaerobic Germination Potential in the BC_1_F_2:3_ Population of Kalarata and NSIC Rc238

QTL NAME	CHR	PEAK MARKER	POS (cM)	INT (cM)	LOD	PVE	ADD
Survivability at 14 DAS							
Screenhouse conditions							
*qSUR3–1*_*Rc238-SCR-14*_	3	SWRm_00276	190.0	178–198	4.90	11.06	13.04
*qSUR5–1*_*Rc238-SCR-14*_	5	SWRm_00427	108.0	104–118	4.50	10.82	−12.47
*qSUR6–1*_*Rc238-SCR-14*_	6	SWRm_00495	36.0	32–46	6.00	14.38	11.66
*qSUR7–1*_*Rc238-SCR-14*_	7	SWRm_01153	62.0	59–66	18.40	36.68	20.13
*qSUR8–1*_*Rc238-SCR-14*_	8	SWRm_00599	66.2	64–74	5.20	12.04	−14.38
Tray-on-table conditions							
*qSUR3–1*_*Rc238-TAB-14*_	3	SWRm_00276	192.0	182–198	5.80	12.79	10.85
*qSUR5–1*_*Rc238-TAB-14*_	5	SWRm_00427	108.0	104–116	4.10	9.76	−13.09
*qSUR6–1*_*Rc238-TAB-14*_	6	SWRm_00495	46.0	38–50	6.60	15.15	9.79
*qSUR7–1*_*Rc238-TAB-14*_	7	SWRm_01153	60.0	58–66	15.30	31.58	17.15
*qSUR8–1*_*Rc238-TAB-14*_	8	SWRm_00599	66.2	64–74	4.90	11.70	−13.14
Survivability at 21 DAS							
Screenhouse conditions							
*qSUR3–1*_*Rc238-SCR-21*_	3	SWRm_00276	194.0	182–202	4.87	11.04	14.03
*qSUR5–1*_*Rc238-SCR-21*_	5	SWRm_00427	110.0	106–116	4.49	10.77	−14.8
*qSUR6–1*_*Rc238-SCR-21*_	6	SWRm_00495	38.0	34–44	5.97	14.28	12.72
*qSUR7–1*_*Rc238-SCR-21*_	7	SWRm_01153	62.0	59–66	20.31	39.66	24.28
*qSUR8–1*_*Rc238-SCR-21*_	8	SWRm_00599	66.2	64–76	5.21	12.03	−16.00
Tray-on-table conditions							
*qSUR3–1*_*Rc238-TAB-21*_	3	SWRm_00276	192.0	182–198	5.55	12.32	11.79
*qSUR5–1*_*Rc238-TAB-21*_	5	SWRm_00427	110.0	106–116	4.57	10.81	−16.90
*qSUR6–1*_*Rc238-TAB-21*_	6	SWRm_00495	44.0	38–48	6.96	16.01	12.11
*qSUR7–1*_*Rc238-TAB-21*_	7	SWRm_01153	62.0	58–66	17.50	35.17	22.76
*qSUR8–1*_*Rc238-TAB-21*_	8	SWRm_00599	66.2	64–74	4.78	11.28	−15.5

*q* indicates QTL, *SCR* screenhouse, *TAB* tray-on-table, *POS* position, *INT* position interval, *LOD* logarithm of odds, *PVE* percent phenotypic variation explained, *ADD* additive effects of the peak marker

**Table 3:** List of QTLs Detected for Anaerobic Germination Potential in the BC_1_F_2:3_ Population of Kalarata and NSIC Rc222

QTL NAME	CHR	PEAK MARKER	POS (cM)	INT (cM)	LOD	PVE	ADD
Survivability at 14 DAS							
Screenhouse conditions							
*qSUR3–1*_*Rc222-SCR-14*_	3	SWRm_00272	10.0	2–12	4.51	10.55	9.88
*qSUR6–1*_*Rc222-SCR-14*_	6	SWRm_00495	135.3	132–136	5.73	13.11	10.30
*qSUR7–1*_*Rc222-SCR-14*_	7	SWRm_01153	60.0	52–64	14.10	29.49	20.70
*qSUR9-1*_*Rc222-SCR-14*_	9	SWRm_00692	18.1	14–28	3.66	9.03	8.22
Tray-on-table conditions							
*qSUR3–1*_*Rc222-TAB-14*_	3	SWRm_00276	2.0	0–12	4.15	9.49	9.27
*qSUR6–1*_*Rc222-TAB-14*_	6	SWRm_00495	135.3	132–136	5.76	13.19	11.77
*qSUR7–1*_*Rc222-TAB-14*_	7	SWRm_01153	60.0	54–64	13.51	28.39	24.52
Survivability at 21 DAS							
Screenhouse conditions							
*qSUR3–1*_*Rc222-SCR-21*_	3	SWRm_00272	10.0	2–12	4.93	11.47	11.16
*qSUR6–1*_*Rc222-SCR-21*_	6	SWRm_00495	135.3	132–136	5.81	13.33	10.71
*qSUR7–1*_*Rc222-SCR-21*_	7	SWRm_01153	60.0	56–64	13.80	28.99	20.01
Tray-on-table conditions							
*qSUR3–1*_*Rc222-TAB-21*_	3	SWRm_00272	10.0	4–14	4.51	10.58	12.31
*qSUR6–1*_*Rc222-TAB-21*_	6	SWRm_00495	135.3	132–136	5.18	11.96	11.95
*qSUR7–1*_*Rc222-TAB-21*_	7	SWRm_01153	60.0	54–64	12.07	25.80	22.11
Control conditions							
*qGER9-1*_*Rc222-CON-21*_	9	SWRm_00679	50.0	46–60	3.67	8.55	−2.90

*q* indicates QTL, *SCR* screenhouse, *TAB* tray-on-table, *POS* position, *INT* position interval, *LOD* logarithm of odds, *PVE* percent phenotypic variation explained, *ADD* additive effects of the peak marker

For the population Kalarata/NSIC Rc238, a total of five significant QTLs on chromosomes 3, 5, 6, 7, and 8 were detected for SUR under both screening conditions (Table 2). Three significant QTLs were detected for SH on chromosomes 1, 3, and 7, of which QTLs on chromosomes 1 and 3 were detected in both screening conditions. The SH QTL on chromosome 3 was also consistent with the QTLs for SUR (Additional file 1: Table S2). A different location on chromosome 7 for SH was detected by screening the population in the screenhouse.

The tolerant parent Kalarata contributed the SUR QTLs *qSUR3–1*_*Rc238-SCR-14*_*, qSUR6–1*_*Rc238-SCR-14*_, and *qSUR7–1*_*Rc238-SCR-14*_ while the QTLs *qSUR5–1*_*Rc238-SCR-14*_ and *qSUR8–1*_*Rc238-SCR-14*_ came from susceptible parent NSIC Rc238 under screen house condition at 14 DAS (Table 2). Same five QTLs showed effect on survival at 14 DAS under tray-on-table condition and at 21 DAS in both screening conditions. The largest effect QTL *qSUR7–1* (named as *qSUR7–1*_*Rc238-SCR-14*_*, qSUR7–1*_*Rc238-TAB-14*_*, qSUR7–1*_*Rc238-SCR-21*_ and *qSUR7–1*_*Rc238-TAB-21*_) showed a LOD score of 15.30 to 20.31 and explained 31.58% to 39.66% of the phenotypic variation, with an additive effect of 17.15% to 24.28%, followed by *qSUR6–1* (named as *qSUR6–1*_*Rc238-SCR-14*_*, qSUR6–1*_*Rc238-TAB-14*_*, qSUR6–1*_*Rc238-SCR-21*_ and *qSUR6–1*_*Rc238-SCR-21*_), which explained 14.28% to 16.01% of the phenotypic variation, detected by a LOD score of 5.97 to 6.96, and had an additive effect of 9.79% to12.72%. *qSUR3–1* (named as *qSUR3–1*_*Rc238-SCR-14*_, *qSUR3–1*_*Rc238-TAB-14*_, *qSUR3–1*_*Rc238-SCR-21*_ and *qSUR3–1*_*Rc238-TAB-21*_) contributed 11.04% to 12.79% of the phenotypic variation, with an additive effect of 10.85% to 14.03%. The SUR QTLs *qSUR5–1* (named as *qSUR5–1*_*Rc238-SCR-14*_, *qSUR5–1*_*Rc238-TAB-14*_, *qSUR5–1*_*Rc238-SCR-21*_ and *qSUR5–1*_*Rc238-TAB-21*_) and *qSUR8–1* (named as *qSUR8–1*_*Rc238-SCR-14*_, *qSUR8–1*_*Rc238-TAB-14*_, *qSUR8–1*_*Rc238-SCR-21*_ and *qSUR8–1*_*Rc238-TAB-21*_) from high-yielding susceptible parent NSIC Rc238 contributed 9.76% to 10.82% and 11.28% to 12.04% of the phenotypic variation, with LOD scores of 4.10 to 4.57 and 4.78 to 5.21, respectively (Table 2, Additional file 1: Figure S2).

All SH QTLs were contributed by Kalarata, the parent with anaerobic germination potential. The largest effect QTL identified for SH is *qSH1–1* (named as *qSH1–1*_*Rc238-SCR-21*_ and *qSH1–1*_*Rc238-TAB-21*_), which is consistent over both screening conditions. It explained a phenotypic variation of 12.5% to 22.1%, with a LOD score of 5.4 to 9.4. The second largest effect QTL detected for SH was consistent with SUR QTL *qSH3–1* (named as *qSH3–1*_*Rc238-SCR-14*_*, qSH3–1*_*Rc238-SCR-21*_ and *qSH3–1*_*Rc238-TAB-21*_*,*), which was also consistent in both screening conditions. This QTL explained phenotypic variation of 10.1% to14.4%, with a LOD score of 3.7 to 5.8 and an additive effect of 0.84% to 5.17%. QTL *qSH7–1*_*Rc238-SCR-21*_ was identified with a LOD score of 4.6 and explained 10.7% of the phenotypic variation and had an additive effect of 4.57%. It was identified only under screenhouse conditions and was located in a different position from the identified QTL (*qSUR7–1*) for SUR (Additional file 1: Table S2, Additional file 1: Figure S3A). Under controlled conditions, no QTL was detected for germination, whereas one QTL on chromosome 1 (*qSH1-2*_*Rc238-CON-21*_) was detected for SH with an LOD score of 6.1 that could explain 14.1% of the phenotypic variation (Additional file 1: Table S2). Altogether, the identified QTLs for SUR explained phenotypic variation in the range of 80.98% to 87.78% while that for the trait SH ranged from 10.1% to 36.5% for the Kalarata/NSIC Rc238 population.

For the population Kalarata/NSIC Rc222, a total of three significant QTLs on chromosomes 3, 6, and 7 were detected for SUR under both screening conditions, which were also consistent with the first population (Table 3). Two significant QTLs were detected for SH on chromosomes 1 and 3, of which the QTL on chromosome 1, detected under both screening conditions, was also common with the first population for the same trait. In all cases, parent Kalarata with anaerobic germination potential contributed the tolerant alleles for AG (Additional file 1: Table S3).

For SUR, the largest effect QTL *qSUR7–1* (named as *qSUR7–1*_*Rc222-SCR-14*_, *qSUR7–1*_*Rc222-TAB-14*_, *qSUR7–1*_*Rc222-SCR-21*_ and *qSUR7–1*_*Rc222-TAB-21*_) was identified by a LOD of 12.07 to 14.10 explaining 25.80% to 29.49% of the phenotypic variation with an additive effect of 20.01% to 24.52%. The second largest effect QTL *qSUR6–1* (named as *qSUR6–1*_*Rc222-SCR-14*_, *qSUR6–1*_*Rc222-TAB-14*_, *qSUR6–1*_*Rc222-SCR-21*_ and *qSUR6–1*_*Rc222-TAB-21*_) explained 11.96% to 13.33% of the phenotypic variation and was detected by a LOD score of 5.18 to 5.81 and had an additive effect of 10.30% to 11.95%. The QTL *qSUR3–1* (named as *qSUR3–1*_*Rc222-SCR-14*_, *qSUR3–1*_*Rc222-TAB-14*_, *qSUR3–1*_*Rc222-SCR-21*_ and *qSUR3–1*_*Rc222-TAB-21*_) explained 9.49% to 11.47% of the phenotypic variation with an additive effect of 9.27% to 12.31% and was detected by a LOD score of 4.15 to 4.93 (Table 3, Fig. 3b).

For SH, the consistent QTL *qSH1–1* (named as *qSH1–1*_*Rc222-SCR-14*_, *qSH1–1*_*Rc222-SCR-21*_ and *qSH1–1*_*Rc222-TAB-21*_) identified by a LOD score of 5.18 to 7.17 had an additive effect of 1.3% to 3.4% and could explain 13.5% to 18.5% of the phenotypic variation. A second position on chromosome 3, *qSH3–2* (named as *qSH3–2*_*Rc222-SCR-14*_ and *qSH3–2*_*Rc222-SCR-21*_) that is different from the detected QTL from the other population for the same trait was identified by an LOD score of 3.78 to 4.87 and could explain 10.2% to 11.7% of the phenotypic variation (Additional file 1: Table S3, Additional file 1: Figure S3B).

Altogether, three stable QTLs on chromosome 3, 6, and 7 for SUR (Fig. 3) and one stable QTL on chromosome 1 for SH (Additional file 1: Figure S3B) were detected using both populations under both screening conditions for 21 DAS, among which the QTLs on chromosome 6 and chromosome 1 have not been reported previously for the traits to the best of our knowledge.

Under control conditions, a QTL for normally established seedlings as germination percentage was detected on chromosome 9 (*qGER9-1*_*Rc222-CON-21*_), which was contributed by susceptible parent NSIC Rc222 (Table 3). The SH QTL on chromosome 1 (*qSH1-2*_*Rc238-CON-21*_) was also detected in a different position in control conditions from the one detected under stress. This QTL was detected by a LOD score of 10.16 and explained 21.9% of the phenotypic variation with an additive effect of 2.3% (Additional file 1: Table S3).

All the identified QTLs explained a total phenotypic variation ranging from 47.34% to 62.18% for SUR and 13.5% to 28.7% for SH in this population.

### Effect of QTL Classes

**Fig. 4:**
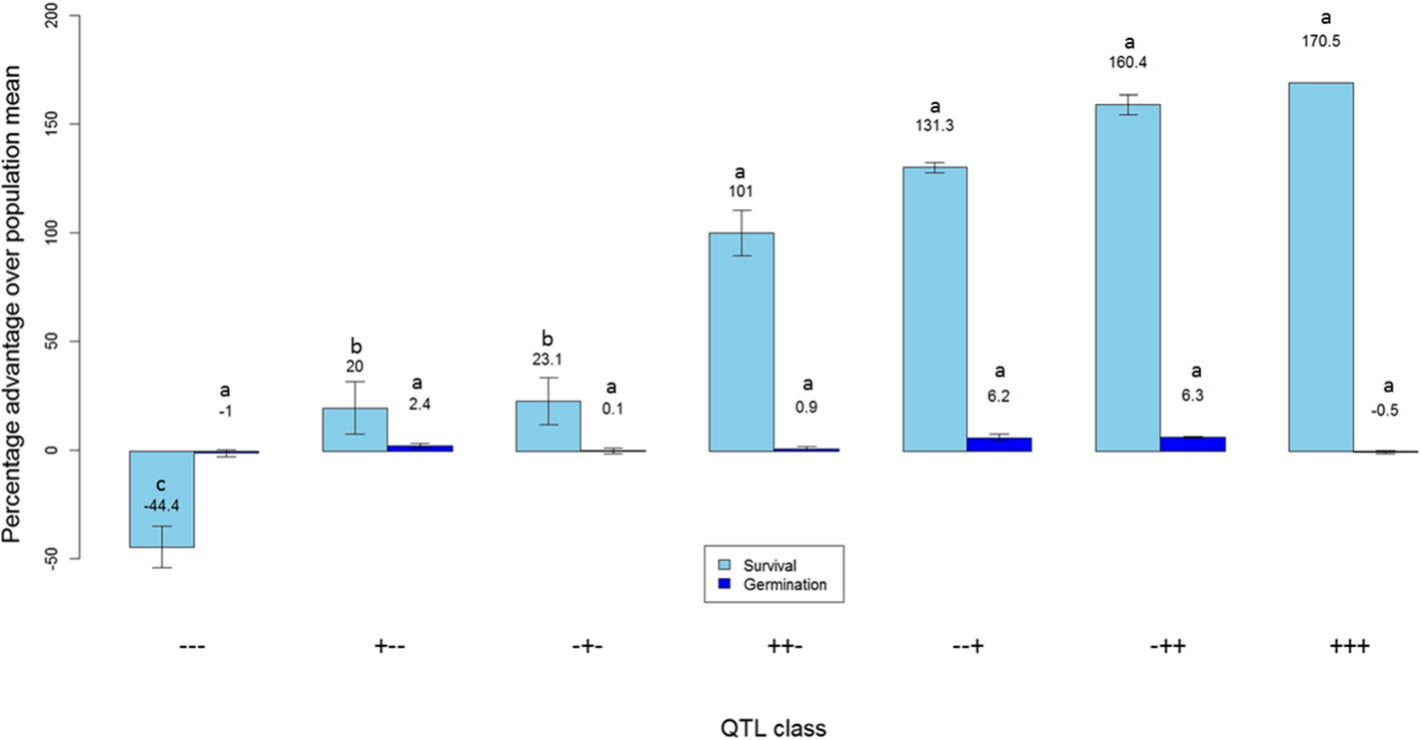
Effect of combinations of *qSUR3–1, qSUR6–1*, and *qSUR7–1* on mean seedling survival under anaerobic conditions at 21 days after seeding. --- refers to lines without the three QTLs, + − - refers to lines with *qSUR3–1*, − + − refers to lines with *qSUR6–1*, ++ − refers to lines with *qSUR3–1* and *qSUR6–1*, −− + refers to lines with *qSUR7–1*, −++ refers to lines with *qSUR6–1* and *qSUR7–1*, and +++ refers to lines with all three QTLs. Letters (“a”, “b” and “c”) above the mean values represent the ranking of each QTL class, where the same letter implies that the mean values are not statistically different

Three QTLs (*qSUR3–1*, *qSUR6–1* and *qSUR7–1*) with a large and stable effect across the two populations were identified in this study. We conducted a combined analysis of the two populations to understand the effect of varying QTL combinations on germination under aerobic and anaerobic conditions at 21 DAS. Figure 4 presents the percentage advantage of varying classes of QTLs over the population mean for both submerged and control conditions. Under control conditions, the three QTLs did not show any significant effect on germination and the advantage of all QTL classes remained close to zero over the population mean as revealed by Pairwise Mann-Whitney U-tests. Under AG conditions, however, *qSUR7–1* showed the highest effect on the trait, followed by *qSUR6–1* and *qSUR3–1*, respectively. It was also observed that lines with *qSUR3–1* and *qSUR6–1* had a significantly higher advantage over the lines with only one of the two QTLs. The QTL *qSUR7–1*, however, provided a high advantage even without the other two QTLs. Its combination with *qSUR6–1* increased the total advantage, which was further increased in the lines with all three QTLs combined, although these are not statistically significant. The increase in advantage of a QTL when combined with the other QTLs indicates their additive nature. Lines with the combination of *qSUR7–1* and *qSUR3–1* were not found in both populations. Moreover, lines without the three QTLs had the lowest germination of all classes which was lower than the population mean.

### Grain Color Analysis and Donor Selection

**Fig. 5:**
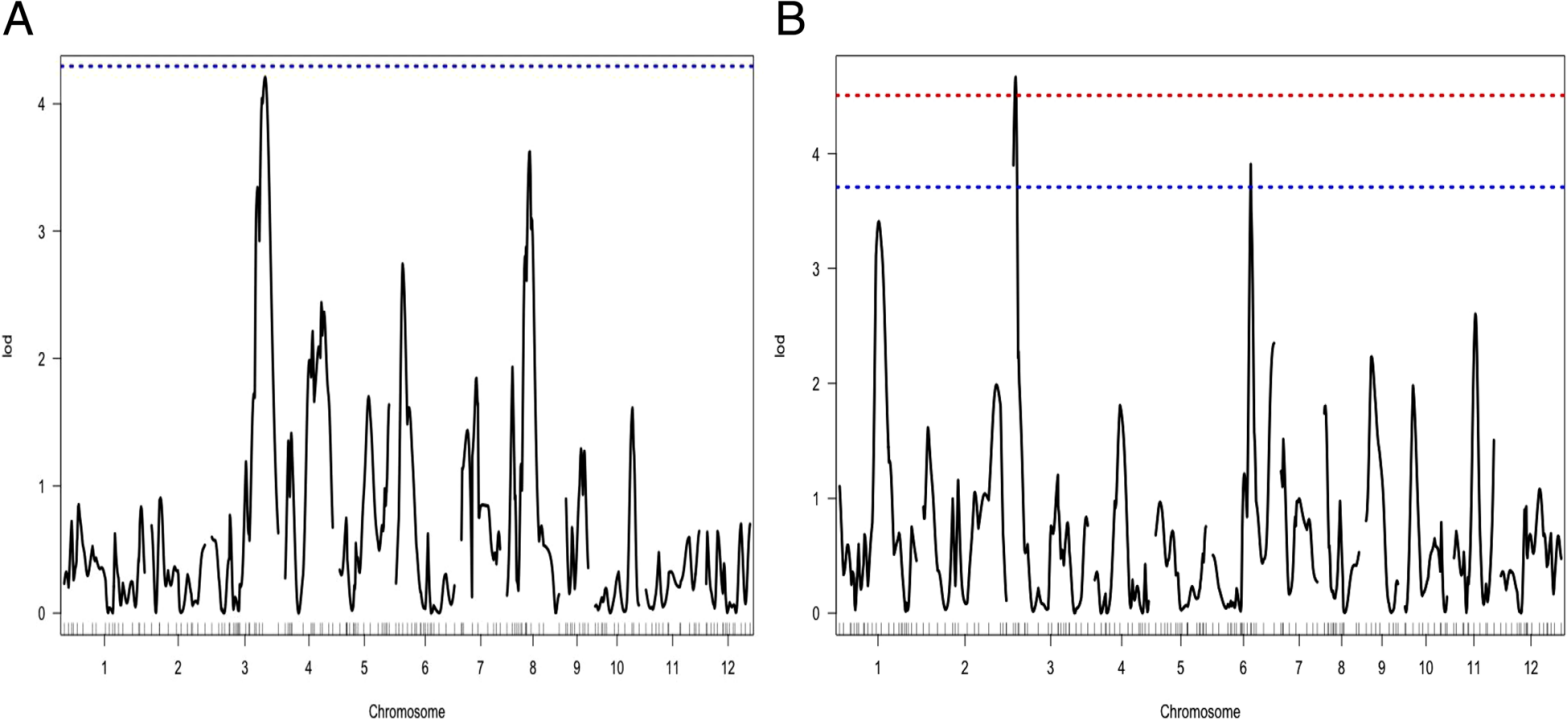
Whole-genome scan plots by interval mapping for survivability at 21 DAS of the white grain-colored lines of the population Kalarata/NSIC Rc238 (**a**) and for the population Kalarata/NSIC Rc222 (**b**) in the screenhouse. Horizontal lines indicate the significant logarithm of odds threshold at 5% and 1% confidence levels (from the bottom to the top) based on 10,000 permutation tests

**Fig. 6:**
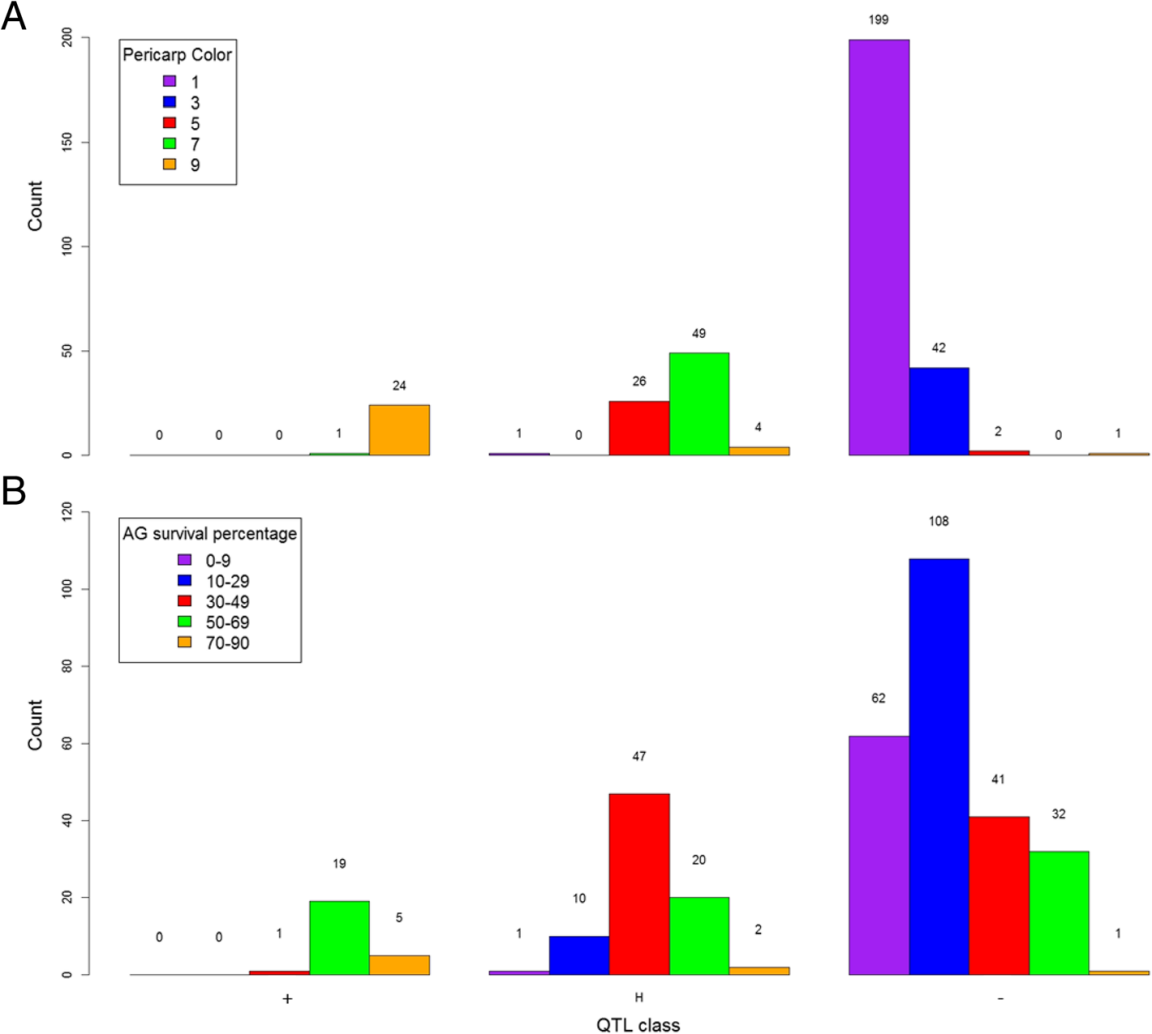
Frequency distribution of pericarp color (**a**) and germination percentage (**b**) within QTL allele classes across the two populations. + refers to lines with Kalarata allele, − refers to lines with Rc222/Rc238 allele, and H refers to heterozygote for the peak marker of qSUR7–1

The tolerant donor (Kalarata) used in this population has a red-colored pericarp. In the past, studies have detected QTLs for high anaerobic germination at chromosome 7, which lie closely to the *Rc* gene that controls pericarp color in rice. QTL mapping for pericarp color revealed a highly significant QTL on chromosome 7 for both populations. The QTLs are located at 52.72 cM and 59.06 cM and accounts for 72.4% and 85.77% phenotypic variation for Kalarata/NSIC Rc222 and Kalarata/NSIC Rc238 populations, respectively (Additional file 1: Table S4). In this study, the largest effect QTL (*qSUR7–1)* was also detected near the said loci at 60 cM and 62 cM for Kalarata/NSIC Rc222 and Kalarata/NSIC Rc238 populations, respectively (Tables 2 and 3). It was important for us to know whether the effect on AG in this study is due to the pleiotropic effect of the *Rc* gene to survival under AG or is due to the effect of other AG-related genes which are in close linkage with the *Rc* gene. A possibility of both phenomena being responsible was also considered. To address this, a separate QTL analysis was conducted among lines with white-colored pericarp. *qSUR7–1* was found non-significant in both populations. *qSUR3–1* (named as *qSUR3–1*_*Rc222-SCR-14*_, *qSUR3–1*_*Rc238-TAB-14*_ and *qSUR3–1*_*Rc238-SCR-21*_) and *qSUR6–1* (named as *qSUR6–1*_*Rc222-SCR-14*_, *qSUR6–1*_*Rc222-TAB-14*_ and *qSUR6–1*_*Rc222-SCR-21*_) were found significant in the Kalarata/NSIC Rc222 population for survivability at 14 and 21 DAS (Fig. 5). However, only *qSUR3–1* (named as *qSUR3–1*_*Rc238-SCR-14*_*, qSUR3–1*_*Rc238-TAB-14*_ and *qSUR3–1*_*Rc238-SCR-14*_) was significant for survivability for the Kalarata/NSIC Rc238 population (Table 4). Also, we conducted analysis to understand the effect of allele classes of *qSUR7–1* peak marker SWRm_01153 on grain pericarp color and survival (Fig. 6a and b). In majority of the cases across both populations, the lines with the Kalarata allele of the QTL had red pericarp (score 7–9) and high AG survival percentage and those without the Kalarata allele had white-colored pericarp and low survival percentage. However, exceptions to this pattern were observed when one line without the Kalarata allele of the QTL showed red-colored pericarp and high AG survival. Similarly, another line segregating for the QTL showed white-colored pericarp. Further analysis revealed that the identified QTL for pericarp color lies within the region of the Rc gene in chromosome 7 at 6.07 Mb while the span of qSUR7–1 varies from this gene. The difference of grain color and survival for three QTL classes indicates a tight linkage between qSUR7–1 and the Rc gene. Correlations between SUR and pericarp color further supported this hypothesis where a wide range of distribution was found for SUR within each color class (Additional file 1: Figure S5). This showed the possibility of independent segregation for the genes underlying the two traits despite the tight linkage.

**Table 4:** List of QTLs Detected for Survivability in the BC_1_F_2:3_ Population of Kalarata and NSIC Rc238 and Kalarata and NSIC Rc222 Using White-Colored Grain

QTL NAME	CHR	PEAK MARKER	POSITION (cM)	PHYSICAL POSITION (Mb)	LOD	PVE	ADD
Kalarata/NSIC Rc238							
Survivability at 14 DAS (Screen house)							
*qSUR3–1*_*Rc238-SCR-14*_	3	SWRm_00276	192.0	36.38	4.41	17.89	16.49
Survivability at 14 DAS (Tray-on-table)							
*qSUR3–1*_*Rc238-TAB-14*_	3	SWRm_00276	192.0	36.38	3.93	16.12	14.18
Survivability at 21 DAS (Screen house)							
*qSUR3–1*_*Rc238-SCR-21*_	3	SWRm_00276	192.0	36.38	4.21	17.14	16.46
Kalarata/NSIC Rc222							
Survivability at 14 DAS (Screen house)							
*qSUR3–1*_*Rc222-SCR-14*_	3	SWRm_00272	8.0	35.39	4.72	19.55	12.70
*qSUR6–1*_*Rc222-SCR-14*_	6	SWRm_00495	135.3	25.03	4.52	18.78	12.09
Survivability at 14 DAS (Tray-on-table)							
*qSUR3–1*_*Rc222-TAB-14*_	3	SWRm_00276	2.0	36.38	4.69	19.43	13.46
*qSUR6–1*_*Rc222-TAB-14*_	6	SWRm_00495	135.3	25.03	4.45	18.55	13.57
Survivability at 21 DAS (Screen house)							
*qSUR3–1*_*Rc222-SCR-21*_	3	SWRm_00272	10.0	35.39	4.67	19.30	13.50
*qSUR6–1*_*Rc222-SCR-21*_	6	SWRm_00495	135.3	25.03	3.90	16.50	12.30

*q* indicates QTL, *LOD* logarithm of odds, *PVE* percent phenotypic variation explained, *ADD* additive effects of the peak marker

We also identified a set of eight lines with different combinations of the three major QTLs identified in this study, white-colored pericarp and high germination under aerobic and anaerobic conditions (Table 5). These lines will provide the basis for developing future populations for further studies on these QTLs and deployment into breeding programs.

**Table 5:** Donor Lines With Varying Combinations of The Three QTLs (in heterozygote condition for *qSUR7–1*), white-colored pericarp, high survival under AG, and germination under control conditions

Line	Population	SUR (%)	Germination (%)	QTL		
				*qSUR3–1*	*qSUR6–1*	*qSUR7–1*
Line 187	NSIC Rc222/Kalarata	50.0	83.0	+	+	–
Line 70	NSIC Rc222/Kalarata	60.0	90.2	–	+	–
Line 145	NSIC Rc222/Kalarata	60.0	87.1	–	–	H
Line 53	NSIC Rc222/Kalarata	63.3	89.3	+	+	–
Line 116	NSIC Rc222/Kalarata	63.3	77.4	+	+	–
Line 12	NSIC Rc238/Kalarata	64.5	82.9	+	+	–
Line 111	NSIC Rc238/Kalarata	56.6	82.5	+	–	–
Line 39	NSIC Rc238/Kalarata	59.1	86.2	+	+	–

+ indicates the presence of a Kalarata allele at the peak marker, − indicates the presence of the NSIC Rc222/NSIC Rc238 allele at the peak marker, H indicates the presence of a heterozygous allele at the peak marker

## Discussion

In this study, we aimed to identify novel and robust genomic regions from the landrace Kalarata that can potentially contribute to further increasing the anaerobic germination potential of existing direct-seeded rice varieties. Two populations derived from the cross of Kalarata with elite recipient lines NSIC Rc222 and NSIC Rc238 were developed and evaluated for germination under different anaerobic screening conditions to identify QTLs stable across genetic backgrounds and across screening methods. In the past, several phenotyping protocols have been considered for screening rice lines for AG. Studies have shown that some rice genotypes can germinate under water and elongate their coleoptile to some extent but ultimately fail to develop roots and shoots. The ability to germinate under water and successfully develop roots and shoots is referred to as SUR. This innate phenomenon is the key trait to mark the genetic variation among the genotypes for several underlying traits that play a role in successful germination under water. Together with SUR, SH is the second most important trait because it reflects fast coleoptile elongation, which is frequently used to study the genetics of anaerobic germination. In our study, the two traits were recorded at 14 and 21 DAS in controlled tray-on-table screening as well as under more field-like conditions in the screenhouse. Apart from this, germination in a controlled environment was also used as a trait to determine early and uniform emergence under non-stress conditions (Fig. 1). This phenotyping approach provides a thorough screening under anaerobic and aerobic conditions and allows the expression of maximum trait variation. The highly significant differences and heritability values achieved in each of the experiments show the robustness of the phenotyping protocols. SUR ranged from 0 to 76.7% and SH ranged from 9.1 to 42.8 cm. It is clear that the ranges of variation of the progenies in both indices of the mapping populations exceeded the parental range (Table 1, Additional file 1: Table S1), indicating transgressive segregation and a contribution from both parents. Septiningsih et al. (2013) also observed similar results for SUR, Hsu and Tung (2015) for coleoptile elongation, and Zhang et al. (2017) for seedling vigor traits. In our study, although most QTLs for SUR were contributed by the tolerant parent Kalarata,13% to 33% of the lines showed lower SUR than the susceptible parent while 14% to 20% of the families showed higher SUR than Kalarata (the donor parent) across the two populations (Additional file 1: Figure S4). This suggests the contribution of genetic factors with small effects and genetic interactions to the higher tolerance of progenies.

The results of the QTL analysis revealed three highly significant QTLs for SUR (Fig. 3) on chromosome 3 (*qSUR3–1*), chromosome 6 (*qSUR6–1*), and chromosome 7 (*qSUR7–1*) across both populations and both screening conditions, explaining a stable expression of phenotypic variation ranging from 47.34% to 64.98%. *qSUR7–1* was the largest effect QTL for SUR, followed by *qSUR6–1* and *qSUR3–1*, respectively. One highly significant QTL on chromosome 1 (*qSH1–1*) for SH was detected in both populations and screening conditions, explaining 12.5% to 22.1% of the phenotypic variation for seedling height. Altogether, the QTLs for the trait SUR explained phenotypic variation ranging from 47.34% to 87.78% while for trait SH it ranged from 10.1% to 36.5%. The other QTLs identified in this study were either population specific or screening condition specific (Tables 2 and 3). The QTLs expressed across genetic backgrounds and screening conditions are key candidates for further validation and pyramiding into other lines to assess their effect across environments and genetic backgrounds and eventual deployment into breeding programs. In order to test the suitability of these QTLs for deployment, we conducted a QTL class analysis to determine the effect of the combination of these three QTLs (Fig. 4). All combinations showed positive additive effects and no negative effects. The highest advantage was observed in lines with the tolerant allele (+) for all three QTLs while those with the susceptible allele (−) at all three loci had the lowest germination percentage. No QTL advantage was observed under control conditions, with all QTL classes showing statistically similar germination percentages. The results clarify the complementary effects of these three QTLs and the suitability of deploying them together for maximum advantage and stability of the traits. The results also showed the uniqueness of these loci in terms of their specificity to AG and no effect was observed under normal conditions. Deployment of these loci along with those underlying fast emergence and early seedling vigor will provide all-around improvement in the germination of rice and lead to higher robustness and stability of the trait. Eight promising donor lines with different QTL combinations, high AG survival, high germination and white-colored pericarp (Table 5) can be used for population development and QTL deployment for further AG breeding. Our study also identified *qSH1–1* for seedling height on chromosome 1 with peak marker at 22.04 Mb (Additional file 1: Tables S2 and S3). This is a novel locus and it can be used for improving seedling height without manipulating the dwarfing gene “*sd1*” region (38.38–38.39 Mb). This trait is crucial not only for DSR and AG but also for stresses such as stagnant flooding, for which taller SH is a desirable trait.

*qSUR7–1* was the largest QTL identified in this study. This QTL co-locates with the *Rc* gene, which is responsible for pericarp color in rice. In the study, a QTL for pericarp color was also identified which coincided with the known position of the Rc gene at 6.07 Mb of chromosome 7 (Sweeney et al., 2006). Since the donor parent Kalarata has a red-colored pericarp while the pericarp color of the two recipients is white, it is important to understand the role of the *Rc* gene in AG. Breeding with this QTL will not be possible if the tolerance of anaerobic conditions during germination is due to the pleiotropic effect of the *Rc* gene. It is possible, however, to work with *qSUR7–1* if it is tightly linked to the *Rc* gene and not pleotropic. In order to test this hypothesis, we conducted another QTL analysis using only lines with white-colored pericarp. No effect of *qSUR7–1* was observed in both populations in this analysis (Fig. 5). Additionally, while testing the seed pericarp color and AG survival percentage of different allele classes of *qSUR7–1* (Fig. 6a and b), we did not find a one-on-one relationship between the traits, where red-colored grains had the Kalarata allele while white-colored grains had the recipient allele in most of the cases. However, exceptions to these were observed in case of SUR, where the distribution of the trait varied across pericarp color classes indicating a tight linkage between qSUR7–1 and the Rc gene. This is further clarified by looking at the correlations between SUR and pericarp color. While, red pericarp lines are generally showing higher survival, a wide range of distribution can be found for SUR within each color class (Additional file 1: Figure S5). Notably, one recombinant line with white pericarp color and high survival percentage was identified and another line with red pericarp color without *qSUR7–1* was also identified (Fig. 6). This provided evidence of a tight linkage between *qSUR7–1* and the *Rc* gene however it still does not rule out the possibility of the role of the *Rc* gene in AG but clarifies that these are separate entities. Further fine mapping is therefore continuing with the identified recombinant lines.

## Conclusions

The present study evaluated two mapping populations derived from the cross of landrace Kalarata with NSIC Rc222 and NSIC Rc238. The populations were screened across two different setups of AG and under control conditions. The study identified three large QTLs with consistent performances across populations and screening conditions. Tolerant alleles for all three QTLs were contributed by Kalarata. Apart from this, another QTL for seedling height was identified on chromosome 1, which showed a consistent performance across populations and screening environments. Although this locus did not show a direct effect of AG, it will be useful in the manipulation of seedling height without using the *sd1* gene. This trait can be useful for obtaining higher seedling vigor under direct-seeded conditions as well as in areas prone to stagnant flooding where taller seedling height is desirable. The three QTLs identified for SUR showed a complementary nature and are suitable for deployment together with other QTLs for seedling vigor and early emergence. The largest QTL, *qSUR7–1*, showed a tight linkage with the *Rc* gene for pericarp color. However, recombinants identified in the study are being used to break this linkage for making use of this QTL. Fine-mapping studies will also indicate the role of the *Rc* gene in SUR under AG in these populations. Lines with different combinations of these QTLs have been identified in this study and will be used for further fine mapping and deployment of these QTLs.

## Additional File

Additional file 1: **Table S1.** Performance of BC_1_F_2:3_ mapping populations of Kalarata/NSIC Rc238 and Kalarata/NSIC Rc222 along with respective parents for seedling height (SH) under anerobic condition. **Table S2.** List of QTLs detected for seedling height in the BC_1_F_2:3_ population of Kalarata and NSIC Rc238. **Table S3.** List of QTLs detected for seedling height in the BC_1_F_2:3_ population of Kalarata and NSIC Rc222. **Table S4.** List of QTLs detected for pericarp color in the BC_1_F_2:3_ mapping populations of Kalarata/NSIC Rc238 and Kalarata/NSIC Rc222. **Figure S1.** Line graph showing the water temperature profile during the experiment period in screen house and on Tray conditions. **Figure S2.** The QTL likelihood curve of LOD score showing peak marker and confidence interval for the trait 21 DAS survivability in Kalarata/NSIC Rc238 population in screenhouse screening conditions. The green line indicates confidence interval while horizontal lines indicate the significant logarithm of odds threshold at 95% and 99% confidence levels (from the bottom to the top) based on 10,000 permutations. The figures on the right show the effect of each of the peak markers. All QTLs are contributed by tolerant parent, Kalarata. **Figure S3.** The QTL likelihood curves of LOD score showing peak marker and confidence interval for the trait 21 DAS seedling height for (A) Kalarata/NSIC Rc238 and (B) Kalarata/NSIC Rc222 in screenhouse screening conditions. The green line indicates confidence interval while horizontal lines indicate the significant logarithm of odds threshold at 95% and 99% confidence levels (from the bottom to the top) based on 10,000 permutations. All QTLs are contributed by tolerant parent, Kalarata. **Figure S4.** Frequency distribution of the survival (SUR) trait for Kalarata/NSIC Rc238 and Kalarata/NSIC Rc222 populations under different screening conditions (screenhouse and tray-on-table) and data collection periods (14 DAS and 21 DAS). Dotted lines refer to the susceptible parent (NSIC Rc 238/NSIC Rc 222) while solid lines refer to the tolerant parent (Kalarata). **Figure S5.** Relation between grain pericarp color and survival under anaerobic condition. (DOCX 560 kb)

## Data Availability

The datasets used and/or analyzed during the current study are available from the corresponding author on request.

## Abbreviations

ADH, EC1.1.1.1: Alcohol Dehydrogenase
AG: Anaerobic Germination
ANOVA: Analysis of Variance
cM: Centimorgan
DAS: Days After Seeding
DNA: Deoxyribonucleic acid
DSR: Direct-seeded Rice
GA: Gibberellic Acid
GER: Germination Percentage
H^2^: Broad-sense Heritability
KASP: Kompetitive Allele Specific PCR
LOD: Logarithm of Odds
MgCl_2_: Magnesium Chloride
QTL: Quantitative Trait Loci
SED: Standard Error of Difference
SH: Seedling Height
SNP: Single Nucleotide Polymorphism
SUR: Survival
