# Supplementary material for: Deciphering Genetics Underlying Stable Anaerobic Germination in Rice: Phenotyping, QTL Identification, and Interaction Analysis
Source: Rice (N Y). 2019 Jul 15;12:50. doi: 10.1186/s12284-019-0305-y (PMC6629739; doi:10.1186/s12284-019-0305-y)
Supplement: Supplementary file 1 — Table S1. Performance of BC1F2:3 mapping populations of Kalarata/NSIC Rc238 and Kalarata/NSIC Rc222 along with respective parents for seedling height (SH) under anerobic condition. Table S2. List of QTLs detected for seedling height in the BC1F2:3 population of Kalarata and NSIC Rc238. Table S3. List of QTLs detected for seedling height in the BC1F2:3 population of Kalarata and NSIC Rc222. Table S4. List of QTLs detected for pericarp color in the BC1F2:3 mapping populations of Kalarata/NSIC Rc238 and Kalarata/NSIC Rc222. Figure S1. Line graph showing the water temperature profile during the experiment period in screen house and on Tray conditions. Figure S2. The QTL likelihood curve of LOD score showing peak marker and confidence interval for the trait 21 DAS survivability in Kalarata/NSIC Rc238 population in screenhouse screening conditions. The green line indicates confidence interval while horizontal lines indicate the significant logarithm of odds threshold at 95% and 99% confidence levels (from the bottom to the top) based on 10,000 permutations. The figures on the right show the effect of each of the peak markers. All QTLs are contributed by tolerant parent, Kalarata. Figure S3. The QTL likelihood curves of LOD score showing peak marker and confidence interval for the trait 21 DAS seedling height for (A) Kalarata/NSIC Rc238 and (B) Kalarata/NSIC Rc222 in screenhouse screening conditions. The green line indicates confidence interval while horizontal lines indicate the significant logarithm of odds threshold at 95% and 99% confidence levels (from the bottom to the top) based on 10,000 permutations. All QTLs are contributed by tolerant parent, Kalarata. Figure S4. Frequency distribution of the survival (SUR) trait for Kalarata/NSIC Rc238 and Kalarata/NSIC Rc222 populations under different screening conditions (screenhouse and tray-on-table) and data collection periods (14 DAS and 21 DAS). Dotted lines refer to the susceptible parent (NSIC Rc 238/NSIC Rc 222) whil [file 12284_2019_305_MOESM1_ESM.docx]

Additional file 1

**Table S1** Performance of BC_1_F_2:3_ mapping populations of Kalarata/NSIC Rc238 and Kalarata/NSIC Rc222 along with respective parents for seedling height

|  | **KALARATA/NSIC Rc238 POPULATION** | | | | | | **KALARATA/NSIC Rc222 POPULATION** | | | | | |
| --- | --- | --- | --- | --- | --- | --- | --- | --- | --- | --- | --- | --- |
|  | **Seedling height 14DAS** | | | **Seedling height 21DAS** | | | **Seedling height 14DAS** | | | **Seedling height 21DAS** | | |
|  | **Control** | **Screen house** | **Tray-on-table** | **Control** | **Screen house** | **Tray-on-table** | **Control** | **Screen house** | **Tray-on-table** | **Control** | **Screen house** | **Tray-on-table** |
| Kalarata | 24.3 | 16.7 | 16.5 | 36.2 | 31.3 | 23.2 | 24.7 | 21 | 11.5 | 34.3 | 25.9 | 27.1 |
| NSIC Rc238/ NSIC Rc222 | 19.1 | 14.3 | 15.4 | 29.4 | 22.0 | 22.0 | 20.5 | 14.1 | 12.4 | 30.8 | 18.9 | 23.9 |
| Mean | 22.8 | 16.5 | 15.6 | 35.5 | 29.7 | 23.7 | 24.0 | 20.2 | 12.9 | 34.3 | 25.3 | 27.4 |
| Range | 16.2-27.8 | 9.1-26.8 | 11.9-19.2 | 28.4-42.8 | 14.1-40.5 | 10.5-36.0 | 13.6-29.8 | 11.6-29.5 | 9.7-18.4 | 20.5-39.9 | 10.0-38.1 | 21.8-35.7 |
| SED | 2.42 | 2.84 | 2.45 | 2.95 | 2.95 | 3.20 | 2.01 | 2.78 | 1.95 | 2.52 | 2.73 | 2.60 |
| P value | **** | **** | **** | **** | **** | **** | **** | **** | **** | **** | **** | **** |
| Heritability | 0.48 | 0.66 | 0.63 | 0.65 | 0.89 | 0.60 | 0.70 | 0.81 | 0.45 | 0.72 | 0.90 | 0.58 |

**** = significant at 0.0001% *P* levels

Table S2 List of QTLs detected for seedling height in the BC_1_F_2:3_ population of Kalarata and NSIC Rc238

| **QTL NAME** | **CHR** | **PEAK MARKER** | **POSITION (cM)** | **CONFIDENCE INTERVAL (cM)** | **LOD** | **PVE** | **ADD** |
| --- | --- | --- | --- | --- | --- | --- | --- |
| **Seedling height at 14DAS** | | | | | | | |
| **Screenhouse conditions** | | | | | | | |
| *qSH3-1_Rc238-SCR-14_* | 3 | SWRm_00256 | 238 | 216-238 | 3.7 | 10.1 | 0.84 |
| **Seedling height at 21DAS** | | | | | | | |
| **Screenhouse conditions** | | | | | | | |
| *qSH1-1_Rc238-SCR-21_* | 1 | SWRm_00052 | 200 | 192-206 | 5.4 | 12.5 | 3.34 |
| *qSH3-1_Rc238-SCR-21_* | 3 | SWRm_00276 | 198 | 192-204 | 4.3 | 10.1 | 5.17 |
| *qSH7-1_Rc238-SCR-21_* | 7 | SWRm_00554 | 80 | 74-84 | 4.6 | 10.7 | 4.57 |
| **Tray-on-table conditions** | | | | | | | |
| *qSH1-1_Rc238-TAB-21_* | 1 | SWRm_00052 | 198 | 194-200 | 9.4 | 22.1 | 2.26 |
| *qSH3-1_Rc238-TAB-21_* | 3 | SWRm_00276 | 210 | 202-222 | 5.8 | 14.4 | 3.42 |
| **Control conditions** | | | | | | | |
| *qSH1-2_Rc238-CON-21_* | 1 | SWRm_01359 | 32 | 28-36 | 6.1 | 14.1 | 2.00 |

*q* indicates QTL, SH indicates seedling height, LOD=logarithm of odds, PVE = percent phenotypic variation explained, ADD=additive effects of the peak marker.

Table S3 List of QTLs detected for seedling height in the BC_1_F_2:3_ population of Kalarata and NSIC Rc222.

| **QTL NAME** | **CHR** | **PEAK MARKER** | **POSITION (cM)** | **INTERVAL (cM)** | **LOD** | **PVE** | **ADD** |
| --- | --- | --- | --- | --- | --- | --- | --- |
|  |  |  |  |  |  |  |  |
| **Seedling height at 14DAS** | | | | | | | |
| **Screenhouse conditions** | | | | | | | |
| *qSH1-1_Rc222-SCR-14_* | 1 | SWRm_00052 | 134 | 128-138 | 7.17 | 18.5 | 2.7 |
| *qSH3-2_Rc222-SCR-14_* | 3 | SWRm_00252 | 48 | 42-82 | 3.78 | 10.2 | 1.8 |
| **Seedling height at 21DAS** | | | | | | | |
| **Screenhouse conditions** | | | | | | | |
| *qSH1-1_Rc222-SCR-21_* | 1 | SWRm_00052 | 134 | 126-142 | 5.71 | 13.5 | 3.4 |
| *qSH3-2_Rc222-SCR-21_* | 3 | SWRm_00252 | 58 | 46-82 | 4.87 | 11.7 | 2.7 |
| **Tray-on-table conditions** | | | | | | | |
| *qSH1-1_Rc222-TAB-21_* | 1 | SWRm_00052 | 129.63 | 126-138 | 5.18 | 13.5 | 1.3 |
| **Control conditions** | | | | | | | |
| *qSH1-2_Rc222-CON-21_* | 1 | SWRm_01359 | 238 | 237.9-240.0 | 10.16 | 21.9 | 2.3 |

*q* indicates QTL, SH indicates seedling height, LOD=logarithm of odds, PVE = percent phenotypic variation explained, ADD=additive effects of the peak marker.

Table S4: List of QTLs detected for pericarp color in the BC_1_F_2:3_ mapping populations of Kalarata/NSIC Rc238 and Kalarata/NSIC Rc222

| **POPULATION** | **CHR** | **PEAK MARKER** | **POSITION (cM)** | **INTERVAL (cM)** | **LOD** | **PVE** | **ADD** |
| --- | --- | --- | --- | --- | --- | --- | --- |
| Kalaratta/NSIC Rc222 | 7 | SWRm_01153 | 52.72 | 52.00-54.00 | 52.84 | 72.4 | 3.866 |
| Kalaratta/NSIC Rc238 | 7 | SWRm_01153 | 59.06 | 59.06-60.00 | 78.32 | 85.77 | 3.886 |

LOD=logarithm of odds, PVE = percent phenotypic variation explained, ADD=additive effects of the peak marker.

**Supplementary Figures**

**Figure S1** Line graph showing the average daily water temperature profile during the experiment period in screen house and on tray conditions.


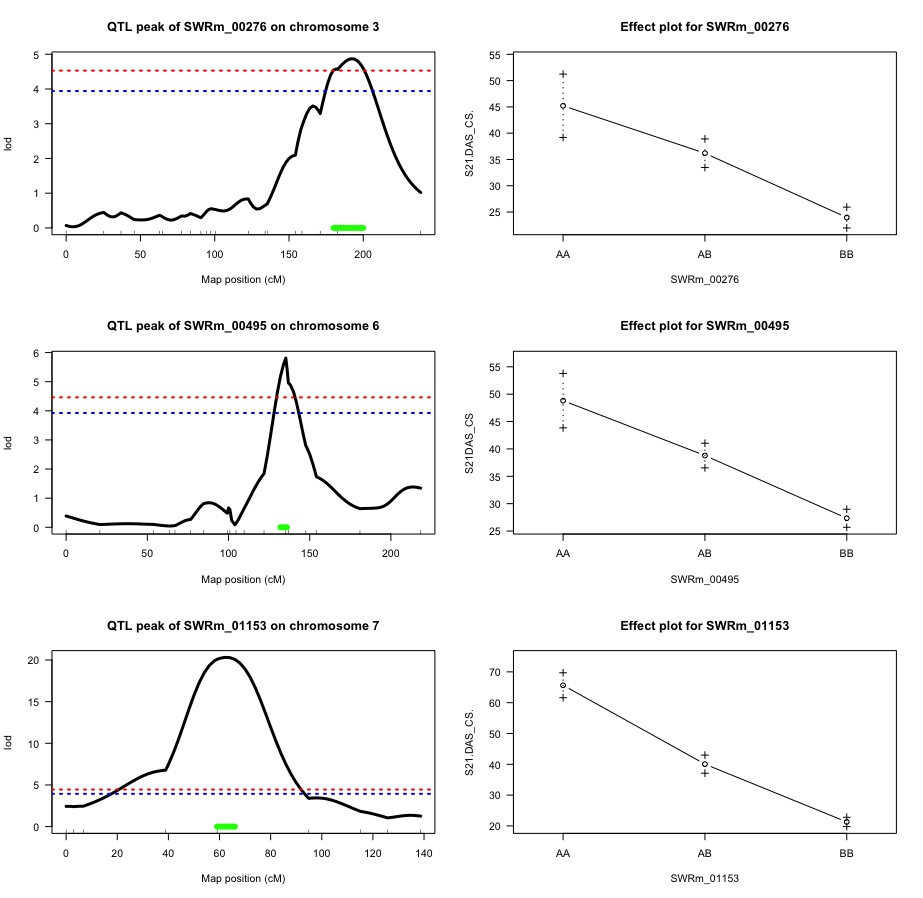


Figure S2 The QTL likelihood curve of LOD score showing peak marker and confidence interval for the trait 21 DAS survivability in Kalarata/NSIC Rc238 population in screenhouse screening conditions. The green line indicates confidence interval while horizontal lines indicate the significant logarithm of odds threshold at 95% and 99% confidence levels (from the bottom to the top) based on 10000 permutations. The figures on the right show the effect of each of the peak markers. All QTLs are contributed by tolerant parent, Kalarata.


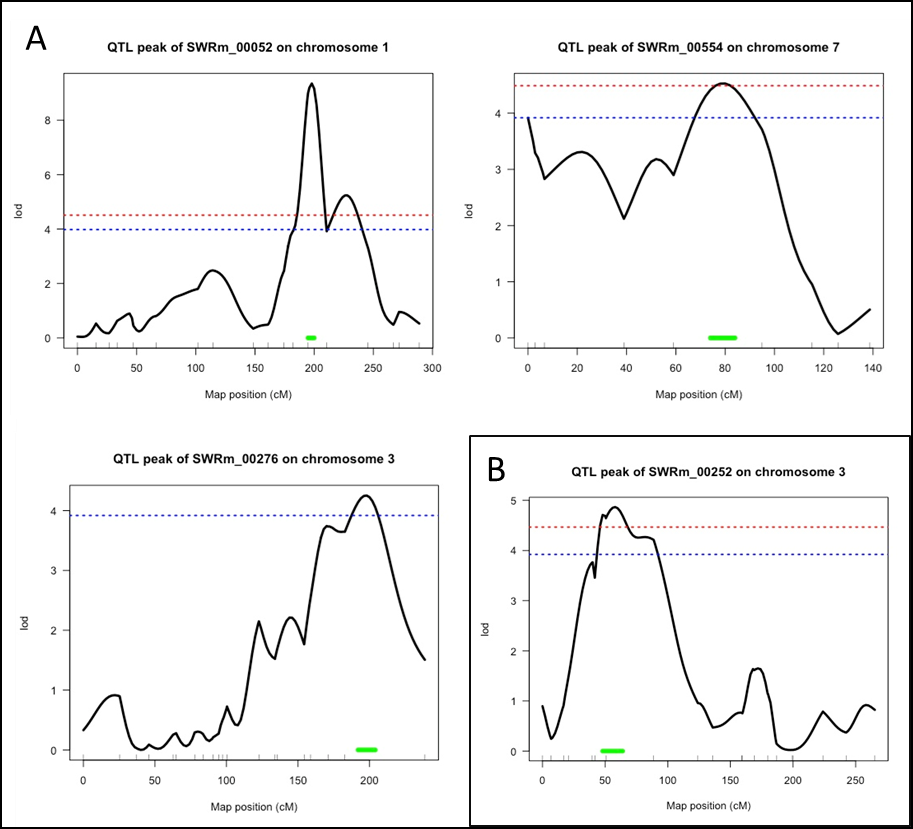


**Figure S3** The QTL likelihood curves of LOD score showing peak marker and confidence interval for the trait 21 DAS seedling height for (A) Kalarata/NSIC Rc238 and (B) Kalarata/NSIC Rc222 in screenhouse screening conditions. The green line indicates confidence interval while horizontal lines indicate the significant logarithm of odds threshold at 95% and 99% confidence levels (from the bottom to the top) based on 10000 permutations. All QTLs are contributed by tolerant parent, Kalarata.


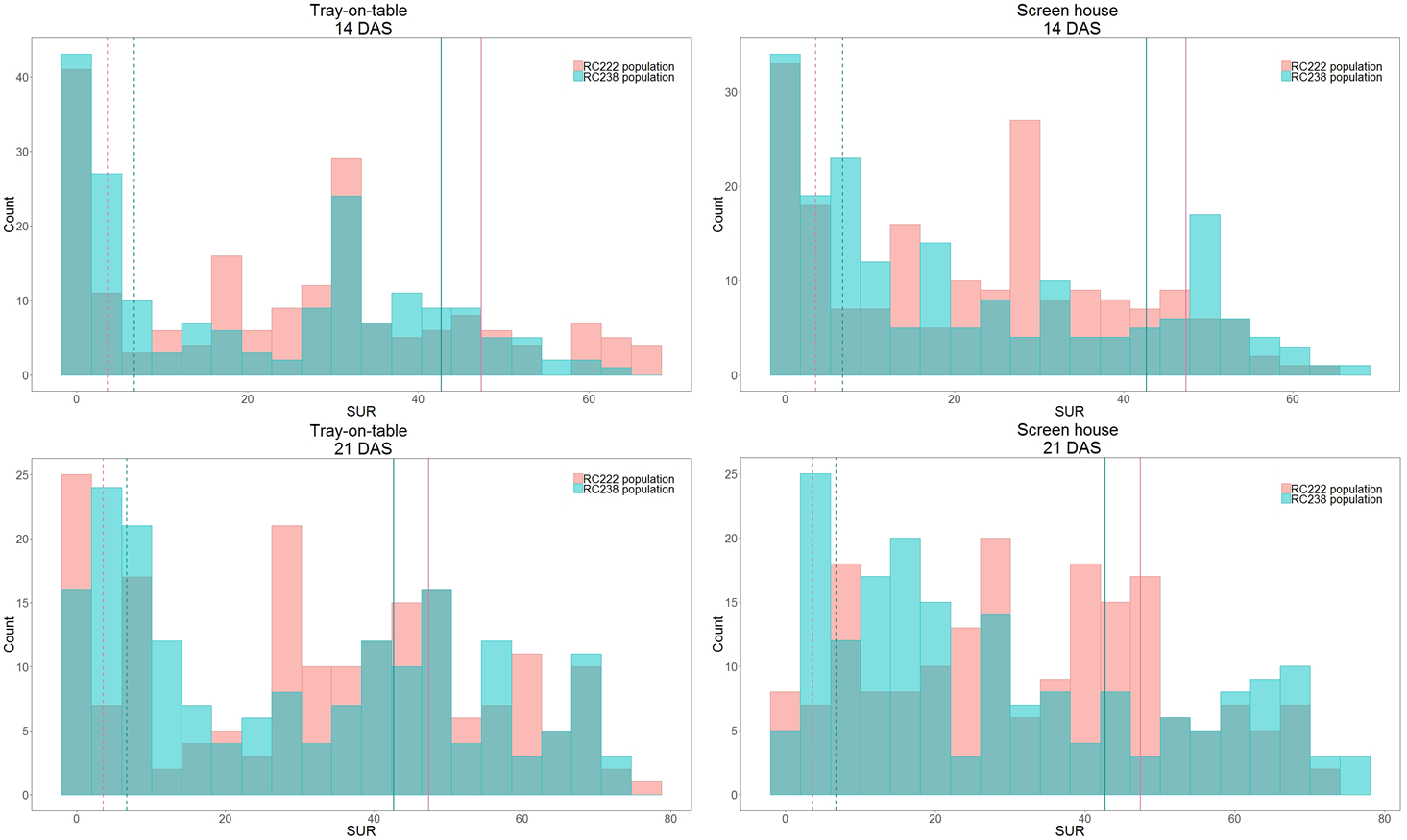


**Figure S4** Frequency distribution of the survival (SUR) trait for Kalarata/NSIC Rc238 and Kalarata/NSIC Rc222 populations under different screening conditions (screenhouse and tray-on-table) and data collection periods (14 DAS and 21 DAS). Dotted lines refer to the susceptible parent (NSIC Rc 238/NSIC Rc 222) while solid lines refer to the tolerant parent (Kalarata).

**Figure S5** Relation between grain pericarp color and survival under anaerobic condition.
